# Evaluation of *NTF4* as a causative gene for primary open-angle glaucoma

**Published:** 2012-06-28

**Authors:** Li Jia Chen, Tsz Kin Ng, Alex H. Fan, Dexter Y.L. Leung, Mingzhi Zhang, Ningli Wang, Yuqian Zheng, Xiao Ying Liang, Sylvia W.Y. Chiang, Pancy O.S. Tam, Chi Pui Pang

**Affiliations:** 1 Department of Ophthalmology and Visual Sciences, the Chinese University of Hong Kong, Hong Kong, China; 2 Prince of Wales Hospital, Hospital Authority, Hong Kong, China; 3 Hong Kong Eye Hospital, Hospital Authority, Hong Kong, China; 4 Joint Shantou International Eye Center of Shantou University and the Chinese University of Hong Kong, Shantou, China; 5 Beijing Tongren Eye Center, Beijing Tongren Hospital, Capital Medical University, Beijing, China

## Abstract

**Purpose:**

The neurotrophin-4 (*NTF4*) gene has been recently implicated in primary open-angle glaucoma (POAG). In this study, we investigated the implication of *NTF4* in POAG among three Chinese cohorts.

**Methods:**

The coding regions and exon-intron boundaries of *NTF4* was sequenced in 950 unrelated Chinese subjects, including a Hong Kong cohort of 390 patients and 230 controls, a Shantou cohort of 130 patients, and a Beijing cohort of 200 patients. Constructs carrying the detected variants were generated using site-directed mutagenesis and transfected into HeLa cells, followed by solubility and migration analyses.

**Results:**

Three variants were identified. p.Pro151Pro was detected in three POAG patients and one control subject. Two novel missense variants, p.Gly157Ala and p.Ala182Val, were identified each in one POAG patient from the Hong Kong cohort, but not in controls. Functional assays showed that the p.Gly157Ala mutant protein was less soluble in Triton X-100, and that migration of HeLa cells transfected with either mutant construct was less than cells transfected with the wildtype.

**Conclusions:**

The *NTF4* variants p.Gly157Ala and p.Ala182Val have been shown to be functional mutations, occurring in 2 of a total of 720 Chinese POAG patients. NTF4 is functionally related to POAG pathogenesis but its mutation frequency is low. Therefore, *NTF4* does not have a major contribution in the molecular genetics of POAG.

## Introduction

Glaucoma is a leading cause of irreversible blindness worldwide. It is characterized by progressive degeneration of retinal ganglion cells and their axons, resulting in visual field defects [1,2]. Primary open-angle glaucoma (POAG) is a major subtype of glaucoma. Elevated intraocular pressure (IOP) is a major risk factor for POAG. According to IOP levels, POAG could be classified into high-tension glaucoma (IOP above 21 mmHg) and normal-tension glaucoma (IOP typically 10 to 21 mmHg) [1]. Nevertheless, both high- and normal-tension glaucoma are considered a continuum rather than separate entities [3].

Genetic risk factors may also play an important role in the mechanisms of POAG. To date, more than 20 linkage loci have been mapped for POAG [4,5], with 3 causal genes, myocilin (*MYOC*; *GLC1A*) [6], optineurin (*OPTN*; *GLC1E*) [7], and WD repeat domain 36 (*WDR36*; *GLC1G*) [8], identified at respective locus. However, each of the genes accounts for only a small proportion (1%–5%) of POAG [5,9-11]. In recent genome-wide association studies, susceptibility variants for POAG were identified at the caveolin 1 (*CAV1*) and caveolin 2 (*CAV2*) [12], transmembrane and coiled-coil domains 1 (*TMCO1*) and CDKN2B antisense RNA 1 (*CDKN2B-AS1*) [13] genes. These genes, however, each has a small to moderate population attributable risk. Further gene mapping and/or validation are warranted.

Recently by screening the neurotrophin-4 (*NTF4*) gene in POAG patients of European origin, Pasutto et al. [14] had identified 7 heterozygous mutations, which altogether accounted for approximately 1.7% of POAG. The *NTF4* gene was later designated as POAG locus - *GLC1O* (OMIM 613100). *NTF4* is located on chromosomal region 19q12–14. It is composed of 2 exons, encoding a protein of 210 amino acids. The NTF4 protein is a member of the neurotrophin protein family associated with the survival of neurons through phosphorylation of tyrosine kinase receptor B (TrkB) receptors. A specific NTF4 signal had been detected in the ganglion cell layer [14]. Moreover, recombinant NTF4 with the most frequent mutation, p.Arg206Trp, caused a decreased activation of TrkB [14]. These findings suggest that the mutant NTF4 proteins might have predisposed to glaucoma via a loss of neurotrophic function. However, the role of *NTF4* in POAG remains controversial. While the identification of a novel mutation in a Singaporean Chinese POAG patient provided positive evidence [15], lack of association was reported in a Caucasian cohort from the United States [16] and an Indian cohort [17].

In this study, we screened the *NTF4* gene in 720 POAG patients from three geographic regions of China: Hong Kong, Shantou and Beijing. Two putative mutations were identified in two patients but not in controls. Subsequent assays suggested that the mutations are functional.

## Methods

### Study subjects

A total of 950 subjects was included in this study: a Hong Kong case-control cohort of 390 POAG patients and 230 controls recruited from the Prince of Wales Hospital and the Hong Kong Eye Hospital, Hong Kong, and two case-only cohorts of 200 POAG patients recruited from Beijing Tongren Hospital and 130 POAG patients from the Joint Shantou International Eye Center (Table 1). Complete ocular examinations were performed for all study subjects. POAG was diagnosed in all recruiting centers on the same criteria: (1) age at diagnosis above 3 years to exclude primary congenital glaucoma; (2) no identifiable primary pathologies for glaucoma, e.g., trauma, uveitis, steroid-induced, exfoliation glaucoma, or neovascular glaucoma; (3) open anterior chamber angle, with Shaffer Grade 2 or above in dark room gonioscopy, without indentation; (4) evidence of characteristic glaucomatous optic disc changes including narrowing of the neuroretinal rim or thinning of the retinal nerve fiber layer; and (5) fulfilling Anderson's criteria for minimal abnormality in glaucomatous visual field [18]. Control subjects were confirmed to have no signs of glaucoma or other major eye diseases, except for mild senile cataract or refractive errors. We purposely recruited control subjects with age 60 years or above, to reduce the chance of detecting disease-related variants in young controls who may develop glaucoma later in life. Age of the Hong Kong control group ranged between 60 and 94 years, with a mean (standard deviation) of 73.5 (7.5) years. This study has been approved by the review boards of all collaborating institutions and performed in accordance with the tenets of the Declaration of Helsinki. Written informed consent was obtained from each subject.

**Table 1:** Demographic and clinical characteristics of the study subjects.

** **	** **	** **	Age at diagnosis (year) *		**Highest recorded IOP, mmHg**	
**Group**	**Sample size**	**Female (%)**	**Range**	**Mean (SD)**	**Range**	**Mean (SD)**
**Hong Kong cohort**						
POAG	390	163 (41.8)	11–88	60.7 (14.8)	10–69	25.3 (9.7)
Control	230	124 (53.9)	60–94	73.5 (7.5)	6–20	13.5 (3.0)
**Shantou cohort**						
POAG	130	27 (20.8)	11–85	46.0 (21.0)	12–58	32.5 (9.6)
**Beijing cohort**						
POAG	200	47 (23.5)	10–82	40.3 (16.8)	12–70	34.6 (12.0)

* For the control subjects, the age refers to the age at study enrolment. IOP: intraocular pressure; POAG: primary open-angle glaucoma; SD: standard deviation.

### Mutation screening of *NTF4* and other glaucoma genes

Table 2 shows the thermal cycler conditions and polymerase chain reaction (PCR) primer sequences, designed referring to the *NTF4* gene sequence from the Ensembl database (accession number ENSG00000167744). The target sequences covering entire coding region and exon-intron boundaries were amplified by PCR and sequenced using the dye-termination chemistry (Big-Dye Terminator Cycle Sequencing Reaction Kit; ver. 3.1; Applied Biosystems, Inc. [ABI], Foster City, CA) on an ABI 3130XL automated sequencer according to the manufacturer’s instructions. Each detected sequence change was confirmed by bidirectional sequencing. We first screened *NTF4* in the bona fide case-control Hong Kong cohort. After the identification of two candidate mutations, we screened the gene in the case-only cohorts from other geographic regions, Shantou and Beijing, to explore more mutations. However, since no further mutation was detected, geographically-matched control subjects of these two cohorts were not analyzed.

**Table 2:** Primer sequences and thermal cycler conditions for *NTF4* sequence analysis.

** **	**Primer sequence**		** **	** **	** **
**Amplifying target**	**Forward primer (5′→3′)**	**Reverse primer (5′→3′)**	**MgCl_2_ (mM)**	**Ta (°C)**	**Size (bp)**
NTF4–1	ACTTGAAGAGGAACTCTGGGAAG	TCAAAACTGCCACTAAGGAGGTA	1.5	58	412
NTF4–2A	CTTCTTTCCCCACTGAAGTTTTT	CACCTTCCTCAGCGTTATCAG	1.5	58	524
NTF4–2B	CCCCGAGTAGTCCTGTCTAGG	CTCTCAGCATCCAGCTCTGTTAT	1.5	58	544

To assess whether the subjects with *NTF4* candidate mutations also carry mutations in glaucoma genes *MYOC*, *OPTN*, and *WDR36*, we screened the coding regions of these three genes in those subjects according to methods described in our previous studies [10,19].

### Cloning of *NTF4*

The open reading frame of *NTF4* (GenBank NM_006179.4) was cloned into an empty pCMV6-AC-IRES-GFP vector between the SgfI and MluI sites (OriGene Technologies, Rockville, MD). Constructs carrying the two missense variants were generated by site-directed mutagenesis (QuikChange Lightning Multi Site-Directed Mutagenesis Kit; Strategene, La Jolla, CA) using specific primers (p.Gly157Ala, sense strand: 5′-CCC GGG GGC AGG TGG AGG GGC CTG CCG GGG AGT GGA CAG GA-3′; p.Ala182Val, sense strand: 5′-CTA TGT GCG GGC ATT GAC CGT TGA TGC CCA GGG CCG TGT GG-3′). All constructs were verified by direct sequencing. The expression of *NTF4* mRNA was confirmed by reverse transcription and PCR, and NTF4 protein expressions detected by immunofluorescence and immunoblotting using monoclonal antibodies against FLAG (Sigma-Aldrich, St. Louis, MO).

### Cell culture and transfection

Human cervical cancer cell line, HeLa (CCL-2; American Type Culture Collection, Manassas, VA) was maintained in Eagle’s Minimal Essential medium (Gibco BRL, Rockville, MD) with 10% fetal bovine serum (Gibco BRL) and 1× penicillin/streptomycin (Gibco BRL) at 37 °C in a humidified incubator containing 5% CO_2_ balanced with air. HeLa cells (10^5^ cells/cm^2^) were transfected with *NTF4* constructs at an optimized ratio of 4 μg constructs to 10 μl transfection reagent (Lipofectamine-2000; Invitrogen, Carlsbad, CA) in Opti-MEM I Reduced Serum medium (Gibco BRL). The *NTF4*-transfected cells were further incubated for 48 h before subsequent analysis. Cells transfected with empty vector were used as control.

### Solubility analysis

Transfected cells were lysed in lysis buffer containing 100 mM Tris-HCl (pH 7.4), 3 mM ethylene glycol tetraacetic acid (Sigma-Aldrich), 5 mM MgCl_2_, 0.5% Triton X-100 (Sigma-Aldrich), 1 mM phenylmethylsulfonylfluoride (Sigma-Aldrich) and protease inhibitor (Roche, Indianapolis, IN). Triton X-100 soluble and insoluble fractions were separately collected for immunoblotting with horseradish peroxidase-conjugated mouse monoclonal antibodies against FLAG (Sigma-Aldrich) and β-actin (Sigma-Aldrich) and detected by enhanced chemiluminescence (Amersham Pharmacia, Cleveland, OH). Triplicates were performed and all three experiments showed similar results.

### Migration analysis

Scratch wounds were created with 200 μl pipette tips on the *NTF4*-transfected cells. Photographs were taken at time 0 (immediately following the scratch wound), 24, and 48 h. The wound gaps were measured by ImageJ (version 1.43u; NIH, Bethesda, MD). The percent migration was calculated by the average area reduction at 24 or 48 h as compared with time 0. Triplicates were performed and all three experiments showed similar results.

### Bioinformatics and statistical analysis

To study the conservation of detected variants across species, we performed multiple sequence alignment of the NTF4 protein by using the clustalw2 program (accessed March 18, 2011). Eight protein sequences from the NCBI or Ensembl databases were used for the alignment, including *Homo sapiens* (human, GenBank NP_006170.1), *Gorilla gorilla* (gorilla, ENSGGOP00000005217), *Pan troglodytes* (chimp, ENSPTRP00000019336), *Macaca mulatta* (monkey, NP_001181760.1), *Pongo pygmaeus* (pongo, ENSPPYP00000011456), *Canis lupus familiaris* (dog, NP_001177358.1), *Rattus norvegicus* (rat; NP_037316.2), and *Mus musculus* (mouse, NP_937833.1).

We then applied two web-based programs to predict deleterious effects of amino acid substitutions in the NTF4 protein. Align-GVGD (accessed March 18, 2011) [20] provides seven discrete grades running from class C0 (most likely neutral) to class C65 (most likely deleterious) for each substitution [21]. When running the program, the multiple sequence alignment generated from the previous step was adopted. The other program, SIFT (accessed March 18, 2011) [22], provides predictions of tolerated and affect protein function. We used the human NTF4 sequence from NCBI (NP_006170.1) as query sequence for the prediction and included all reported variants in the analyses. Based on the predictions from Align-GVGD and SIFT, we categorized the variants and compared the frequency of each category or combined categories between cases and controls using Fisher’s exact test. To supplement the solubility analysis, we predicted the hydrophobicity of NTF4 proteins (wild type and mutant) by using ProtScale (accessed March 18, 2011) [23]. We predicted secondary protein structure using NetSurfP (version 1.1; accessed March 18, 2011) [24].

## Results

### Variants detected in *NTF4*

**Figure 1:**
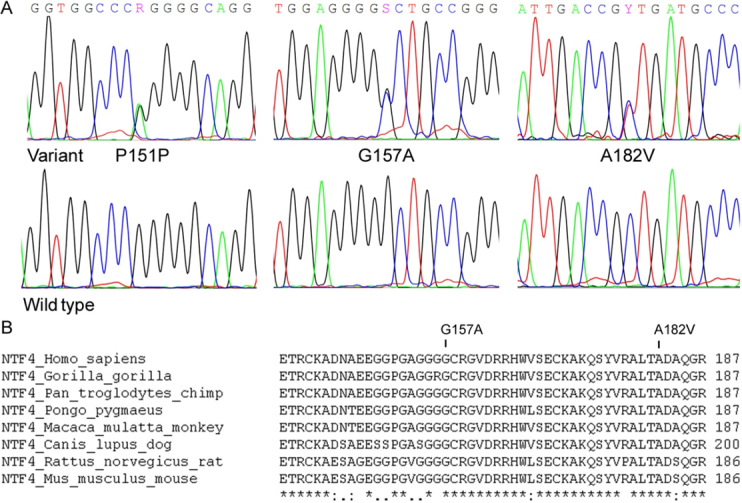
DNA chromatograms and multiple amino acid sequence alignments of detected *NTF4* variants. **A**: DNA chromatograms of the three *NTF4* variants detected in this study. **B**: Multiple amino acid sequence alignments show evolutionary conservation of the mutated residues among mammalian species.

A total of three heterozygous variants, c.453G>A (p.Pro151Pro), c.470G>C (p.Gly157Ala) and c.545C>T (p.Ala182Val; Figure 1A), were detected in the Hong Kong and Beijing cohorts. No sequence change was detected in the Shantou cohort. The synonymous change p.Pro151Pro has been reported [17], while p.Gly157Ala and p.Ala182Val were novel. p.Pro151Pro was detected in 2 patients and 1 control subject of the Hong Kong cohort, and in 1 patient of the Beijing cohort. It is unlikely to be pathogenic as it does not cause amino acid substitution and is present in a control subject. The two missense variants, p.Gly157Ala and p.Ala182Val, were detected in two respective patients in the Hong Kong cohort. p.Gly157Ala was found in a male patient with bilateral normal-tension POAG, diagnosed at 67 years of age with the highest recorded IOP being 17 mmHg in both eyes. p.Ala182Val occurred in a female patient with bilateral high-tension POAG. The age at diagnosis was 28 years and the highest recorded IOP were 36 and 40 mmHg in the right and left eye, respectively. Both patients had typical glaucomatous visual field changes and no known family history of glaucoma. However, their family members were not available for clinical and genetic assessments. Both variants were absent in the control subjects. The affected amino acid positions are highly conserved among orthologs in primates, dog, mouse, and rat (Figure 1B). If p.Gly157Ala and p.Ala182Val are genuinely pathogenic, the *NTF4* gene would account for approximately 0.51% (2/390; 95% CI: 0.14%–1.85%) of POAG in the Hong Kong Chinese population. However, when all Chinese patients were combined, the occurrence would be 2 in 720, i.e., 0.28%.

### Summarizing analysis of *NTF4* variants

Reported *NTF4* variants with respective glaucoma phenotypes of the carriers and functional predictions by Align-GVGD and SIFT are shown in Table 3. A total of 20 coding variants have been identified in 62 individuals out of 4,515 study subjects, including 2 synonymous, 1 nonsense, and 17 missense changes. Sixteen variants were detected in Caucasians [14,16], 2 in Indians [17], and 4 in Chinese from the study of Vithana et al. [15] and this present study. Except for p.Ala88Val, p.Pro151Pro and p.Arg206Trp, each of the remaining 17 variants was found only in one study cohort. Moreover, each of these 17 variants was detected in a single case or control subject, except p.Ser89Asn. Regarding the carriers, 30 were patients with POAG, 3 with primary angle-closure glaucoma (PACG), and 29 were healthy or population controls. By using SIFT, 10 missense variants were predicted to affect protein function while 7 tolerated. With Align-GVGD, one missense change (p.Thr207Ser) was graded as class C0, indicating most likely neutral, and 9 as class C65 indicating most likely deleterious. Another 1, 2, 1, 1, and 2 missense variants were graded as class C15, C25, C35, C45, and C55, respectively.

**Table 3:** Distribution of *NTF4* variants in different study populations.

** **	** **	** **	**European [****14****]**		**American [****16****]**		**Indian [****17****]**			**Chinese [****15****] and this study**	
**Variant**	**Align- GVGD**	**SIFT**	**POAG**	**Control**	**POAG**	**Control**	**POAG**	**PACG**	**Control**	**POAG**	**Control**
p.Cys7Tyr	Class C65	APF	1	0	-	-	-	-	-	-	-
p.Ser29Stop	-	-	-	-	0	1	-	-	-	-	-
p.Glu84Lys	Class C55	Tolerated	1	0	-	-	-	-	-	-	-
p.Ala88Val	Class C65	APF	5	0	1	5	3	3	14	-	-
p.Ser89Asn	Class C45	APF	-	-	1	1	-	-	-	-	-
p.Arg90His	Class C25	Tolerated	1	0	-	-	-	-	-	-	-
p.Arg90Cys	Class C65	APF	-	-	0	1	-	-	-	-	-
p.Leu113Ser	Class C65	APF	-	-	-	-	-	-	-	1 †	0
p.Arg114Gly	Class C65	Tolerated	-	-	0	1	-	-	-	-	-
p.Arg133His	Class C25	APF	-	-	1	0	-	-	-	-	-
p.Arg140Cys	Class C65	APF	-	-	0	1	-	-	-	-	-
p.Pro151Pro	-	-	-	-	-	-	1 *	0	0	3 *, ‡	1 *, ‡
p.Gly157Ala	Class C55	Tolerated	-	-	-	-	-	-	-	1 ‡	0
p.Ala182Val	Class C65	Tolerated	-	-	-	-	-	-	-	1 ‡	0
p.Asp196Asp	-	-	0	1 *	-	-	-	-	-	-	-
p.Arg206Trp	Class C65	APF	5	0	1	2	-	-	-	-	-
p.Arg206Gln	Class C35	APF	1	0	-	-	-	-	-	-	-
p.Thr207Ile	Class C15	Tolerated	-	-	1	0	-	-	-	-	-
p.Thr207Ser	Class C0	Tolerated	0	1	-	-	-	-	-	-	-
p.Arg209Gly	Class C65	APF	1	0	-	-	-	-	-	-	-
Total	-	-	15/892	1/895	5/443	12/533	3/141	3/111	14/285	3/894	0/321

* The synonymous variant *p.Pro151Pro* was not counted in the total frequency. † Variant detected in the study of Vithana et al. [15]. ‡ Variant detected in our study. APF: “affects protein function” predicted by SIFT; PACG: primary angle-closure glaucoma; POAG: primary open-angle glaucoma.

Regarding the distribution of variants between cases and controls, no individual variant showed a statistically significant difference (data not shown). The most common variant, *p.Ala88Val*, was present slightly more in controls (1.1%, 19/1,713) than in patients (0.8%, 12/1,587) in the combined Caucasian and Indian samples, being not associated with glaucoma (p=0.37). It was absent in 1215 Chinese subjects. The second most common variant, p.Arg206Trp, was found in 6 patients (0.45%) and 2 controls (0.14%) in two Caucasian cohorts, but the frequencies were not significantly different (p=0.17). It was not detected in the Chinese or Indian cohorts. When p.Ala88Val was excluded, any missense changes were present in 17 patients (0.69%) and 7 controls (0.34%), with no significant difference (p=0.15; Table 4). Moreover, when the variants were stratified by Align-GVGD grades or SIFT predictions, no individual or combined category was significantly more frequent in cases than in controls (Table 4). Therefore, results of our analysis did not support a statistical link between *NTF4* variants and POAG causality.

**Table 4:** Analysis of missense variants, stratified by Align-GVGD grades and SIFT predictions.

**Stratification**	**Cases (%)**	**Controls (%)**	**p-value §**
**Align-GVGD**			
Class C0	0 (0)	1 (0.05)	0.45
Class C15	1 (0.04)	0 (0)	1.0
Class C25	2 (0.08)	0 (0)	0.50
Class C35	1 (0.04)	0 (0)	1.0
Class C45	1 (0.04)	1 (0.05)	1.0
Class C55	2 (0.08)	0 (0)	0.50
Class C65 *	10 (0.40)	5 (0.25)	0.44
Classes C0 to C45	5 (0.20)	2 (0.10)	0.47
Classes C55 to C65 *	12 (0.48)	5 (0.25)	0.23
Classes C0 to C65 *	17 (0.69)	7 (0.34)	0.15
p.Ala88Val	12 (0.48)	19 (0.93)	0.073
Non-carrier	2452 †	2007 ‡	ref
**SIFT**			
Affect protein function *	12 (0.48)	5 (0.25)	0.23
Tolerated	5 (0.20)	2 (0.10)	0.47
Non-carrier	2452 †	2007 ‡	ref

* The most common variant p.Ala88Val was not included. † Including patients with POAG and PACG. ‡ Excluding the carrier with the nonsense variant p.Ser29Stop. § p-values were estimated with Fisher’s exact test.

### Functional analysis of *NTF4* variants

**Figure 2:**
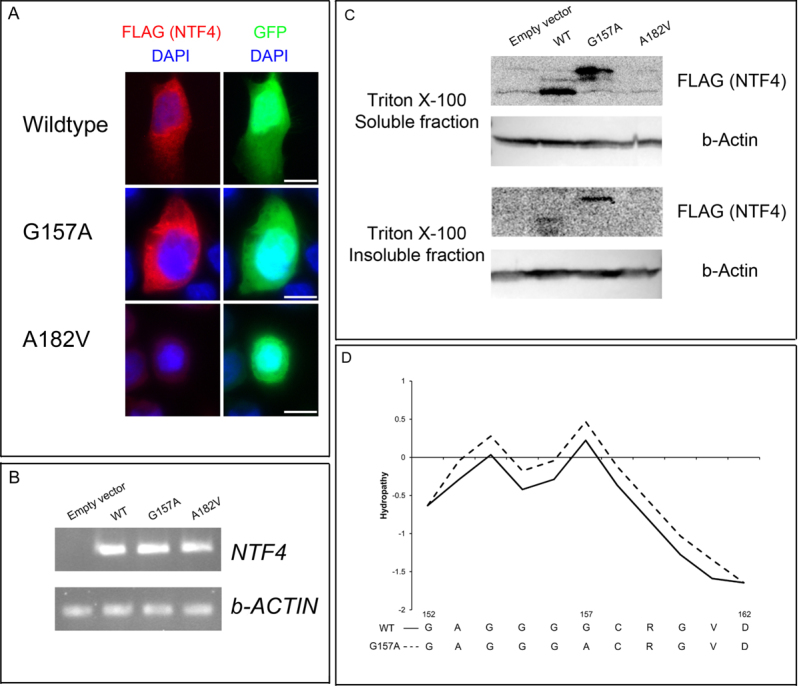
Functional assays of the two *NTF4* mutations. **A**: Immunofluorescence analysis of *NTF4*-transfected HeLa cells. *NTF4*-transfected HeLa cells were fixed with 3% paraformaldehyde and stained with mouse monoclonal antibodies against FLAG. The immunofluorescence pattern of NTF4 wildtype protein with FLAG tag suggested cytoplasmic localization. The pattern of p.Gly157Ala variant protein showed no difference with that of wildtype. However, p.Ala182Val variant protein could not be detected despite the indication of GFP signal for a transfected cell. Scale bar: 20 μm. **B**: Gene expression analysis of *NTF4*-transfected HeLa cells. Total RNA of the *NTF4*-transfected cells was collected, extracted and reverse transcribed. *NTF4* and β-actin (*ACTB*) gene expressions were detected by polymerase chain reaction. All *NTF4*-transfected cells, but not empty vector-transfected cells, expressed *NTF4* transcript. **C**: Solubility analysis of NTF4 proteins. *NTF4*-transfected HeLa cells were lysed by the Triton X-100 lysis buffer. The soluble supernatant and insoluble pellets were analyzed separately by immunoblotting using horseradish peroxidase-conjugated mouse monoclonal antibodies against FLAG and β-actin. NTF4 wildtype and p.Gly157Ala variant proteins were expressed and soluble in Triton X-100 buffer. However, p.Gly157Ala variant protein was less soluble than wildtype. The p.Ala182Val variant protein could not be detected in both fractions. **D**: Hydrophobicity analysis of NTF4 proteins. The hydrophobicity of NTF4 amino acid sequences was predicted by ProtScale. Around the position 150, the signal in p.Gly157Ala was elevated compared to that in wild type (WT), suggesting increase in hydrophobicity of the variant.

**Figure 3:**
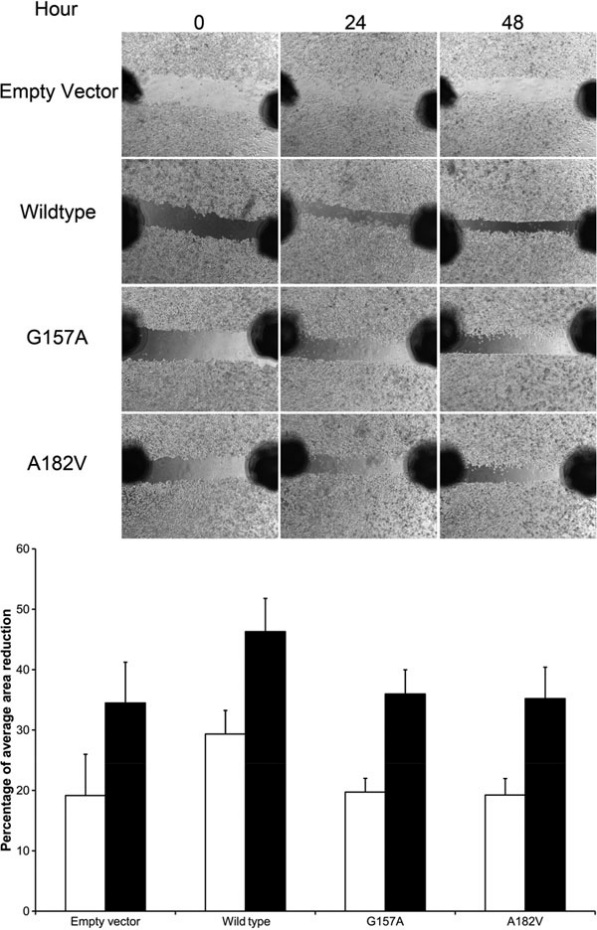
Migration analysis of NTF4 proteins. Scratch wounds were created with 200-μl pipette tips on the *NTF4*-transfected HeLa cells. The medium was replaced by fresh serum-free medium. Photomicrographs were taken at time 0, 24, and 48 h. The wound gaps were measured, and the percent migration was calculated by the average area reduction of 24 or 48 h as compared with time 0. The average area reduction in HeLa cells expressing NTF4 wild type protein was larger than that in cells expressing NTF4 variants at hour 24 and 48.

The functional effects of the two missense variants were studied by in vitro assays. Only the p.Gly157Ala, but not the p.Ala182Val protein, could be detected by immunoblotting (Figure 2A), although wildtype NTF4 and both mutant transcripts were detected after transfection (Figure 2B). Wildtype NTF4 and the p.Gly157Ala mutant proteins were both present in Triton X-100 soluble fraction. However, the p.Gly157Ala mutant protein was less soluble in Triton X-100 and observed in the insoluble fraction (Figure 2C). This might be due to an increase in hydrophobicity predicted by ProtScale (Figure 2D) and reduction of coil probability by NetSurfP. Moreover, similar immunofluorescent patterns were found between the wildtype and p.Gly157Ala mutant proteins (Figure 2A), suggesting that the amino acid change would not affect protein trafficking. Furthermore, impaired function in the NTF4 variants could be observed from the migration analysis. HeLa cells transfected with the NTF4 mutant constructs migrated less than those transfected with the wild type construct (Figure 3). These results indicate that the NTF4 variants affect the protein properties.

## Discussion

Results in this study show that *NTF4* is less polymorphic in Chinese than in Caucasians. We have identified two novel putative mutations, p.Gly157Ala and p.Ala182Val, among 720 patients with POAG. They were absent in 460 chromosomes from healthy subjects. With the exception of p.Pro151Pro, we found no previously reported variant such as the most common variant p.Ala88Val in Caucasians and p.Leu113Ser detected in a Chinese patient in Singapore [15]. In our in vitro assays, the p.Gly157Ala mutant protein was less soluble in Triton X-100. The HeLa cells transfected with either mutant construct showed less migration when compared to those with the wildtype construct. Moreover, both p.Gly157Ala and p.Ala182Val caused an increased hydrophobicity of the NTF4 protein and a reduced cell migration, suggesting that the mutations could have compromised the protein functions and subsequently cellular mobility. In this study, p.Ala182Val was identified in a patient with high-tension glaucoma while p.Gly157Ala was found in a patient with normal-tension glaucoma. Therefore, NTF4 may be involved in pathways affecting ganglion cells viability, rather than playing a role in IOP elevation. If these two mutations are genuinely pathogenic, the *NTF4* gene would account for approximately 0.5% of POAG in Hong Kong Chinese, similar to that in Singaporean Chinese (0.57%) [15]. However, no mutation was found in the Shantou and Beijing cohorts. Therefore the *NTF4* gene is not likely to have a major contribution in the genetic epidemiology of POAG.

So far, the role of *NTF4* in POAG remains controversial. While the results of Pasutto et al. [14], Vithana et al. [15] and our study supported its implication in POAG, other reports showed a lack of association [16,17]. Notably, the two most common mutations *p*.Ala88Val and p.Arg206Trp detected by Pasutto et al. [14] was over-represented in control subjects in the studies of Liu et al. and Rao et al. [16,17]. Moreover, Liu et al. [16] had detected some variants in controls only (see Table 3). Furthermore, results of our summarizing analysis showed that no individual or group of mutations was significantly associated with POAG. Therefore, it is likely that a *NTF4* mutation alone is not sufficient to explain glaucoma pathogenesis, and other factors should have played a role. In fact, it is not uncommon that certain disease-causing mutations occur in healthy subjects of different ethnicities. For example, a variant *p.Glu121Lys* in the nuclear receptor subfamily 2, group E, member 3 (*NR2E3*) gene was causative for enhanced S-cone syndrome in Caucasian [25], whereas it was found in more than 10% of Chinese normal subjects [26]. Likewise in glaucoma, common pathogenic mutations had been found in unaffected individuals suggesting an incomplete penetrance, such as the *MYOC* p.Thr377Met [27-30], *OPTN* p.Glu50Lys [31], and *WDR36* p.Asp658Gly [8,32]. Therefore it is likely that pathogenic mutations in these genes, including *NTF4*, would cause glaucoma together with other factors, such as the general genetic backgrounds, gene risk variants and environmental risk. Further support to this view is the occurrence of the p.Leu113Ser mutation in a patient with unilateral POAG [15]. Additional local risk factors might have acted, additively or interactively, with mutant NTF4 in this patient to cause glaucoma. To examine whether mutations in *MYOC*, *OPTN*, and *WDR36* played a role, Vithana et al. [15] screened the 3 genes in the patient but found no mutation. Similarly, we detected no mutation in *MYOC*, *OPTN*, and *WDR36* in the two patients with *NTF4* mutations.

In genetic studies, segregation analysis provides direct measures of disease susceptibility. However, it was not available in all reported mutational screening studies of *NTF4* in POAG. Thus, we applied Align-GVGD and SIFT to predict the functional impacts of all reported missense variants and compared their distributions between cases and controls, with or without stratification by the Align-GVGD grades and SIFT predictions. However, no mutation was found to be significantly associated with POAG.

In summary, we have identified two novel variants, p.Gly157Ala and p.Ala182Val, in the *NTF4* gene. Functional assays showed that the mutant NTF4 proteins have altered properties and may compromise cell functions. Our findings show that NTF4 is functionally related to POAG pathogenesis. However, the mutation frequency of *NTF4* is low in overall Chinese patients, suggesting that it does not contribute a major role in the molecular genetics of POAG.
